# Cyclic stretching boosts microRNA‐499 to regulate Bcl‐2 via microRNA‐208a in atrial fibroblasts

**DOI:** 10.1111/jcmm.16373

**Published:** 2021-02-18

**Authors:** Su‐Kiat Chua, Bao‐Wei Wang, Ying‐Ju Yu, Wei‐Jen Fang, Chiu‐Mei Lin, Kou‐Gi Shyu

**Affiliations:** ^1^ School of Medicine College of Medicine Fu Jen Catholic University New Taipei Taiwan; ^2^ Division of Cardiology Department of Internal Medicine Shin Kong Wu Ho‐Su Memorial Hospital Taipei Taiwan; ^3^ Department of Emergency Medicine Shin Kong Wu Ho‐Su Memorial Hospital Taipei Taiwan

**Keywords:** adult human atrial fibroblasts, aortocaval shunt, B‐cell lymphoma 2, cycling stretch, microRNA‐208a, microRNA‐499

## Abstract

MicroRNAs that modulate transcription can regulate other microRNAs and are also up‐regulated under pathological stress. MicroRNA‐499 (miR‐499), microRNA‐208a (miR‐208a) and B‐cell lymphoma 2 (Bcl‐2) play roles in cardiovascular diseases, such as direct reprogramming of cardiac fibroblast into cardiomyocyte and cardiomyocyte apoptosis. Whether miR208a, miR499 and Bcl‐2 were critical regulators in cardiac fibroblast apoptosis under mechanical stretching conditions in human cardiac fibroblasts‐adult atrial (HCF‐aa) was investigated. Using negative pressure, HCF‐aa grown on a flexible membrane base were cyclically stretched to 20% of their maximum elongation. In adult rats, an aortocaval shunt was used to create an in vivo model of volume overload. MiR208a was up‐regulated early by stretching and returned to normal levels with longer stretching cycles, whereas the expression of miR499 and Bcl‐2 was up‐regulated by longer stretching times. Pre‐treatment with antagomir‐499 reversed the miR‐208a down‐regulation, whereas Bcl‐2 expression could be suppressed by miR‐208a overexpression. In the HCF‐aa under stretching for 1 h, miR‐499 overexpression decreased pri‐miR‐208a luciferase activity; this inhibition of pri‐miR‐208a luciferase activity with stretching was reversed when the miR‐499‐5p binding site in pri‐miR‐208a was mutated. The addition of antagomir‐208a reversed the Bcl‐2‐3′UTR suppression from stretching for 1 h. Flow cytometric analysis revealed that pre‐treatment with miR‐499 or antagomir‐208a inhibited cellular apoptosis in stretched HCF‐aa. In hearts with volume overload, miR‐499 overexpression inhibited myocardial miR‐208a expression, whereas Bcl‐2 expression could be suppressed by the addition of miR‐208a. In conclusion, miR‐208a mediated the regulation of miR‐499 on Bcl‐2 expression in stretched HCF‐aa and hearts with volume overload.

## 1 INTRODUCTION

A microRNA is a small, non‐coding strand of RNA with approximately 22 nucleotides, which functions in RNA silencing by interacting with the 3′‐untranslated regions of the target mRNA that result in the destabilizing effects of target mRNA (a process referred to as gene silencing).^1^ This results in the target RNA being silenced through one or more of the following processes: cleavage of the mRNA strand, destabilization of the mRNA by reducing the length of its poly(A) tail, or less efficient translation of the mRNA into proteins by ribosomes.^2^ MicroRNAs (miRNA) are known to be widely involved in gene regulation in the pathogenesis of cardiovascular diseases, including cellular hypertrophy, fibrosis and apoptosis of cardiomyocytes.^3^ Global miRNA expression profiling has identified miRNA‐499 (miR‐499), miRNA‐208a (miR‐208a) in the heart, and these miRNAs play important roles in cardiomyocyte hypertrophy and fibrosis in response to stress.^4^, ^5^

Like other RNAs, miRNA is also a product of transcription; therefore, one miRNA may regulate the expression of another miRNA.^6^, ^7^ For miR499, approximately 30% of its indirect cardiac mRNA target regulation is due to higher‐order effects from secondarily regulated microRNAs.^6^ The miR‐208a regulation by miR‐499 expanded the number of miR‐499‐modulated cardiac microRNAs.^6^ However, van Rooij et al observe miR499 down‐regulation upon miR208a knock down in their study.^5^ Such microRNA‐mediated regulation of microRNA can be detected when properly assayed.

B‐cell lymphoma 2 (Bcl‐2), encoded in humans by the *BCL2* gene, is the founding member of the Bcl‐2 family of regulator proteins, which are key regulatory components of the mitochondrial cell death pathways.^8^ Bcl‐2 and its family members, such as Bcl‐xl, Bcl‐w and Mcl‐1, are cytoprotective; other family members, such as Bad, Bak, Bax, Bid, Bim and Bmf, promote apoptosis.^9^ Bcl‐2 is localized in the outer membrane of mitochondria where it plays an important role in promoting cellular survival and inhibiting the actions of proapoptotic proteins.^9^, ^10^

The proliferation of atrial fibroblasts under pathological conditions plays an important role in atrial myocardial electrical and morphological remodelling. Several experimental studies have reported that atrial cardiac fibroblasts couple electrically with myocytes through gap junctions, exerting an electrotonic influence and causing atrial fibrillation.^11^, ^12^ Cyclic mechanical stretching of cultured cells (ie subjecting the cells to repeated stretching and relaxation) at all levels comparable with dynamic stretch overload in vivo has been well studied, including studies that investigated the molecular mechanisms of gene expression and signal transduction in cardiomyocytes, vascular smooth muscle cells and neonatal rat cardiomyocytes.^13^, ^14^, ^15^ However, the effect of mechanical stretching on atrial cardiac fibroblasts has rarely been reported.

The relationship between miR‐499, miR‐208a and Bcl‐2 in stretched atrial cardiac fibroblast and the haemodynamically overloaded heart has not been clearly established. Additionally, the molecular regulatory mechanisms underlying miR‐499‐ and miR‐208a‐induced apoptosis of stretched atrial cardiac fibroblasts remain poorly understood. Therefore, the present study aimed to investigate the molecular mechanism of regulating miR‐499 and miR‐208a on Bcl‐2 protein expression in stretched atrial fibroblasts and in a rat model of volume overload‐induced heart failure.

## 2 MATERIALS AND METHODS

### 2.1 Primary culture of HCF‐aa

HCF‐aa, obtained from ScienCell Research Laboratories (San Diego, CA, USA), were cultured in a fibroblast growth medium (Catalog #2301, ScienCell Research Laboratories, CA, USA) containing essential and non‐essential amino acids, vitamins, organic and inorganic compounds, hormones, growth factors, trace minerals and a low concentration (2%) of foetal bovine serum. The medium was buffered with HEPES and bicarbonate and had a pH of 7.4 when equilibrated in a humidified incubator containing 5% CO_2_ at 37°C.

### 2.2 In vitro cyclic stretching of cultured HCF‐aa

The Flexcell FX‐2000 strain unit (Flexcell International) consists of a vacuum unit linked to a valve controlled by a computer program. HCF‐aa cultured on a flexible membrane base was subjected to cyclic stretching by applying a sinusoidal negative pressure, and the peak negative pressure was 15 kPa. A 20% elongation cyclic stretch was performed at a frequency of 1 Hz (60 cycles/min) for various periods of time as previously description.^15^

### 2.3 Rat model aortocaval shunt

As mentioned previously, aortocaval (AV) shunts were placed in adult Wistar rats to induce volume overload.^16^ Polyethylene catheters and a Grass tachograph preamplifier were used for haemodynamic monitoring. At the end of the shunt period (which lasted for a various number of weeks, according to the protocol), the rats were killed with an excess of isoflurane. Tissues were obtained from the left ventricle for Western blot analysis and miRNAs quantification as previously described.^17^ All animal procedures were conducted according to our Institutional Committee of Animal Care and Use (protocol no. 0990816001) and conformed to the guidelines in the Guide for the Care and Use of Laboratory Animals published by the US National Institutes of Health (publication no. 86‐23, revised 1996).

### 2.4 Quantitative analysis of the microRNAs and mRNA

To quantify miRNA transcripts, RNA eluate was subjected to reverse transcription (RT) with random primer using miScript II RT Kit (QIAGEN^®^, Hilden, Germany). Primers used for miR499, miR208a and U6 were purchased from QIAGEN^®^ with Catalog numbers MS00004375 (5'‐UUAAGACUUGCAGUGAUGUUU‐3’), MS00045829 ( 5'‐GAGCUUUUGGCCCGGGUUAUAC‐3’) and MS00033740, respectively. The working concentration of primer is 0.5 uM. The procedure was performed with miScript SYBR^®^ Green PCR Kit (QIAGEN^®^, Hilden, Germany) according to the manufacturer's instructions.

As for mRNA quantification, total RNA was extracted by TRzol® Reagent (Cat no.15596018, Invitrogen, USA). The RNA was subjected to reverse transcription (RT) with High‐Capacity cDNA Reverse Transcription Kit (REF no.4368813 ABI by Themo Fisher Scientific, USA). Primers used for human Bcl2 and α‐tubulin were obtained from SIGMA® and the sequences as follows: human Bcl2 5′‐GATTGTGGCCTTCTTTGAG‐3′ (forward) and 5′‐GTTCCACAAAGGCATCC‐3′ (reverse); α‐tubulin 5′‐GATCACCAATGCTT GCTTTGAG‐3′ (forward) and 5′‐ACCATGGCGAGGGTCACAT‐3′ (reverse). The working concentration of primer is 1μm, and the procedure was performed with Fast SYBR® Green Master Mix (REF no.4385612, ABI by Themo Fisher Scientific, USA) according to the manufacturer's instructions.

### 2.5 Construction and delivery of miR‐499 and miR‐208a antagomirs and mutant microRNA expression vectors into cultured HCF‐aa and ventricular myocardium

The 85‐bp hsa‐miR‐499 precursor construct was generated as follows: First, genomic DNA was amplified with the forward primer CACGCCCTCTGCAGGC and reverse primer CAGGACTCCCTCCCATGG. The 200 bp amplified product was digested with *Eco*RI and *Bam*HI restriction enzymes and ligated into a pmR‐ZsGreen1 plasmid vector, which coexpressed miR‐499 and green fluorescent protein (Clontech Laboratories, Mountain View, CA, USA). Then, the miR‐499 antagomir and mutant miR499 precursor (Applied Biosystems) construct were designed and ligated into the same plasmid vector as miR‐499.

A 71 bp rat‐miR‐208a precursor construct was generated similarly. Genomic DNA was amplified with forward primer CAACAGAAGTGCTTGGAAG and reverse primer GGCTGATCGACGGTAGCT. The 165 bp amplified product was digested with *Eco*RI and *Bam*HI restriction enzymes and ligated into a pmR‐ZsGreen1 plasmid vector, which coexpressed miR‐208a and green fluorescent protein (Clontech Laboratories, Mountain View, CA, USA). Then, the miR‐208a antagomir and mutant miR‐208a precursor construct (Applied Biosystems) were designed and ligated into the same plasmid vector as miR‐208a.

The constructed plasmids were transfected into the HCF‐aa using a low pressure‐accelerated gene gun (Bioware Technologies, Taipei, Taiwan), essentially according to the manufacturer's instructions. Briefly, 2 mg plasmid DNA was suspended in 5 ml phosphate‐buffered saline and delivered to cultured HCF‐aa or left ventricular myocardium under a helium pressure of 15 psi. A dissecting fluorescence microscope with a high‐resolution CCD camera (Hamamatsu Photonics, Hamamatsu, Japan) was used to visualize the distribution of the treated HCF‐aa or myocardium. In the rat AV shunt model, the chest cavity was reopened 3 days later, and a fluorescent image of the left ventricular myocardium was obtained to determine whether the transfection was successful. The results indicated that the transfection efficiency is 30%.

### 2.6 RNA interference

The HCF‐aa were transfected with 800 ng Bcl‐2 siRNA (Sigma, Singapore). According to a computer program provided by Dharmacon, Bcl‐2 siRNAs are target‐specific 19‐nucleotide siRNAs. The base sequences targeted by Bcl‐2 siRNA were as follows: sense, 5′‐AUGCAAGUGAAUGAACACC‐3′ and 5′‐GGUGUUCAUUCACUUGCAU‐3′. The Bcl‐2 scramble siRNA was used as a negative control, with the following base sequences: sense, 5′‐AUUGUUGGACUUUCGAUCC‐3′ and 5′‐GGAUCGAAAGUCCAACAAU‐3′. After overnight incubation, cells were stretched and analysed by Western blot and quantitative analysis of microRNAs.

### 2.7 Luciferase activity assay

A luciferase reporter assay was introduced to verify the relationship between miR‐499‐5p and pri‐miR‐208a and between miR‐208a‐3p and Bcl‐2‐3′UTR, in the HCF‐aa under mechanical stretching. To construct reporter vectors bearing the miR‐499‐5p target site, we synthesized 400bp fragments that contained the miR‐499‐5p target site (AGATGCCACTGCAGGTAGGGA) and miR‐499‐5p binding mutant site (TGTTGCGTGACGTGCAAGGGT) at the pri‐miR‐208a. The amplified product was ligated into pmirNanoGLO luciferase vectors (Promega) with Sac I and Xba I restriction enzymes. In addition, a 500 bp human Bcl‐2‐3′‐UTR DNA fragment was also generated through artificial synthesis. The Bcl‐2‐3′‐UTR contained miR‐208a‐3p conserved sites at 3′‐UTR (from 3542 to 3563 bp). For the mutant, the conserved site ACAAGATGGTAATCCGACTTAT was mutated into TGTTCAAGGAAATCGCAGAATA and constructed using the same method. All the cloned plasmids were confirmed through DNA sequencing (Seeing Bioscience Co. Ltd. Taipei, Taiwan). The test plasmid (2 μg) was transfected with the gene gun in each well and then replaced with a normal culture medium. Following various periods of mechanical stretch exposure, cell extracts were prepared using the Nano‐Glo dual‐luciferase reporter assay system (Promega) and measured for luciferase activity by using a luminometer (Glomax Multi Detection System, Promega, Madison, WI, USA).

### 2.8 Protein isolation

The HCF‐aa subjected to stretching were harvested by scraping and then centrifuged at 300 g for 10 min at 4°C. The pellet was resuspended and homogenized in modified RIPA buffer, including 1× PBS, 0.3% SDS, 0.5% sodium deoxycholate and 1% NP‐40, by centrifuging at 10,600 g (centrifugal force) for 20 min. A Bio‐Rad protein assay was used to quantify the protein content of supernatant. Equal amounts of protein (30 μg) were loaded into a 10% sodium dodecyl sulphate (SDS)‐polyacrylamide minigel and then electrophoresed.

### 2.9 Western blot analysis

As described previously, the HCF‐aa or ventricular cardiomyocytes were homogenized in a modified RIPA buffer. Equal amounts of protein (30 μg) were loaded into a 10% SDS‐polyacrylamide minigel and then electrophoresed. The protein samples were mixed with a sample buffer, boiled for 10 min, separated by SDS‐polyacrylamide gel electrophoresis under denaturing conditions, and then electroblotted onto nitrocellulose membranes. The blots were incubated overnight in Tris‐buffered saline containing 5% milk to block the non‐specific binding of the antibody. Proteins of interest were revealed by adding specific antibodies as indicated (at 1:200 dilution) and incubating for 1 h at room temperature, followed by incubation for 1 h at room temperature with a 1:10,000 dilution of horseradish peroxidase‐conjugated polyclonal anti‐goat antibody. In this study, Bcl‐2 antibody (sc‐7382, Santa Cruz Biotechnology, Inc) and anti‐alpha tubulin antibody (T 5168, Sigma, USA) were used. Chemiluminescence detection was used to visualize the signals. The equal load of protein in the samples was verified by staining with the monoclonal antibody of alpha tubulin. Densitometry was used to quantify Western blots.

### 2.10 Immunofluorescence staining of cultured HCF‐aa

Cell samples are harvested and treat with 4% paraformaldehyde for 1hr at room temperature (RT). The samples are washing three times with PBS then incubated with primary antibody at 4°C for 12h. Washing three times with PBS again. Incubate with fluorescence‐conjugated secondary antibody in PBS for 1‐2 hours at RT in the dark. Then stained with DAPI to display the nuclei. Finally, mount coverslip and examine using fluorescence microscopy. Each sample is taken at least three random fields and compared with the average number of positive cells.

### 2.11 Flow cytometric analysis for quantifying apoptosis

Apoptotic cells were quantified as the percentage of cells with hypodiploid (sub‐G1) DNA. HCF‐aa were fixed with 70% ethanol and treated with RNase. The nuclei were then stained with propidium iodide, 50ug/ml, Molecular Probes, Eugene, OR, USA and fluorescein isothiocyanate‐annexin V (FITC Annexin V Apoptosis Detection Kit 1, Cat No.: 556547, BD Pharmingen^TM^, USA) The HCF‐aa negative for both annexin V and propidium iodide were still alive, whereas those that were positive for annexin V and negative for propidium iodide were undergoing apoptosis. The HCF‐aa that were positive for both annexin V and propidium iodide is at the end stage of apoptosis, which is known as second apoptosis. A FACSCalibur flow cytometer with Cell Quest software (Becton Dickinson, Franklin Lakes, NJ, USA) was used to measure DNA content. All the assays counted 10,000 cells.

### 2.12 Statistical analysis

The results are expressed as mean ± standard deviation. Analysis of variance and post hoc Tukey‐Kramer multiple comparison tests (GraphPad Software Inc., San Diego, CA, USA) were used for statistical significance. *P *< .05 was considered statistically significant.

## 3 RESULTS

### 3.1 Mechanical stretching inhibited miR‐208a in human cardiac fibroblasts‐adult atrial but enhanced miR‐499 and Bcl‐2 mRNA and protein expression

**FIGURE 1:**
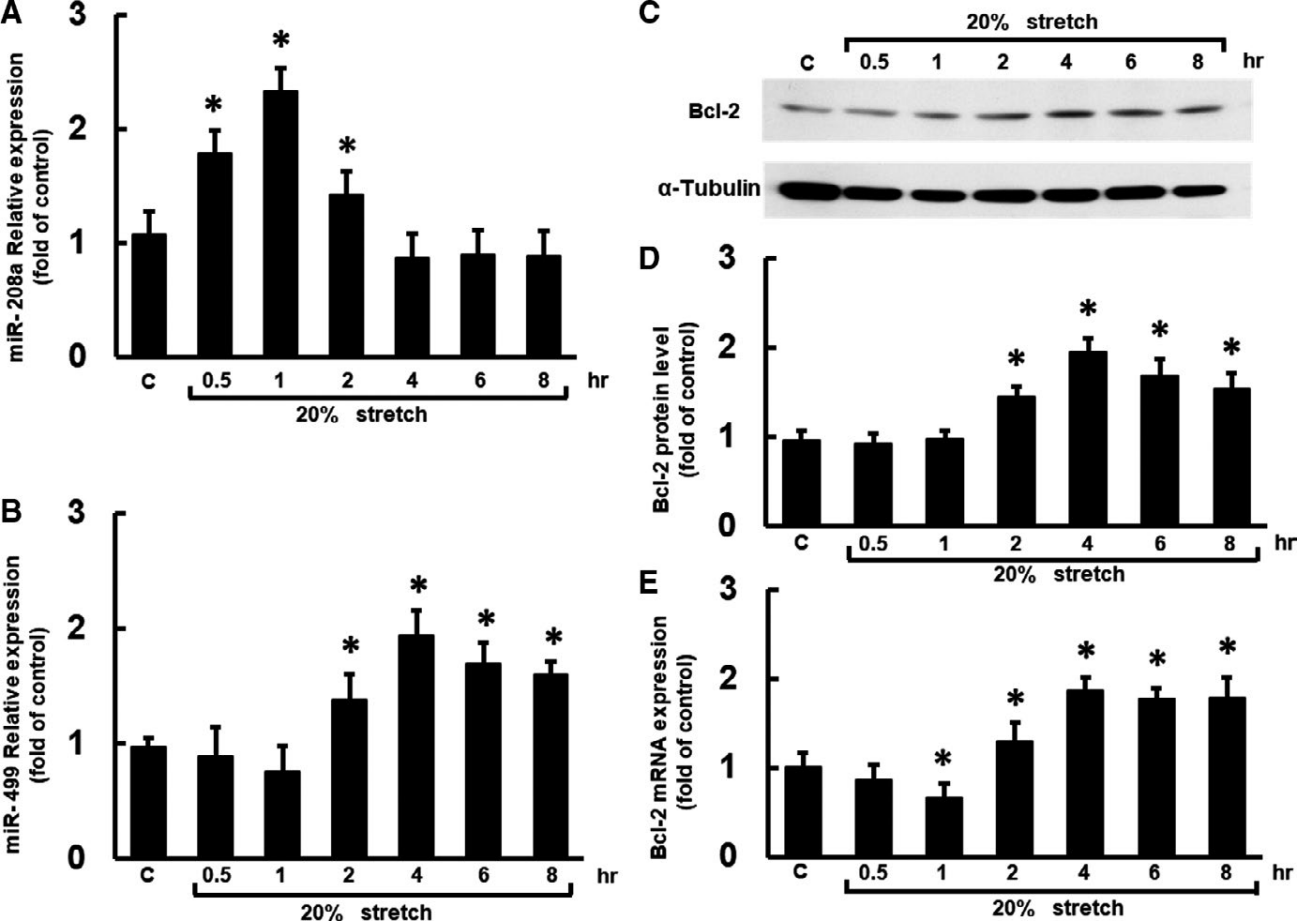
Mechanical stretching inhibited miR‐208a in human cardiac fibroblasts‐adult atrial (HCF‐aa) but enhanced miR‐499, Bcl‐2 mRNA and Bcl‐2 protein expression. A, B, Relative expression levels of miR‐208a and miR‐499 in the HCF‐aa subjected to 20% mechanical stretching for 0‐8 h. C, Representative Western blot for Bcl‐2 protein in the HCF‐aa subjected to 20% stretching for 0‐8 h. D, Quantitative analysis of Bcl‐2 protein levels. The values from the stretched HCF‐aa were normalized to control cell values. E, Bcl‐2 mRNA expression in the HCF‐aa subjected to 20% stretching for 0‐8 h. (n = 3 per group) *P < .001 vs. control

Real‐time quantitative polymerase chain reaction (PCR) was used to measure miR‐208a and miR‐499 levels to examine the effect of mechanical stretching on miR‐208a and miR‐499 in human cardiac fibroblasts‐adult atrial (HCF‐aa). Mechanical stretching with 20% elongation for 0.5‐2 h resulted in significantly higher miR‐208a expression than in the control cells; however, stretching for 4‐8 h restored miR‐208a expression to control levels (Figure 1A). Mechanical stretching for 2‐8 h resulted in significantly higher miR‐499 (Figure 1B). Besides, the Bcl‐2 mRNA expression at stretching for 1 h was significantly lower than control, followed by Bcl‐2 mRNA and protein up‐regulated at mechanical stretching for 2‐8 h (Figure 1C–E).

### 3.2 MiR‐499 decreases pri‐miR‐208a luciferase activity in stretched HCF‐aa

**FIGURE 2:**
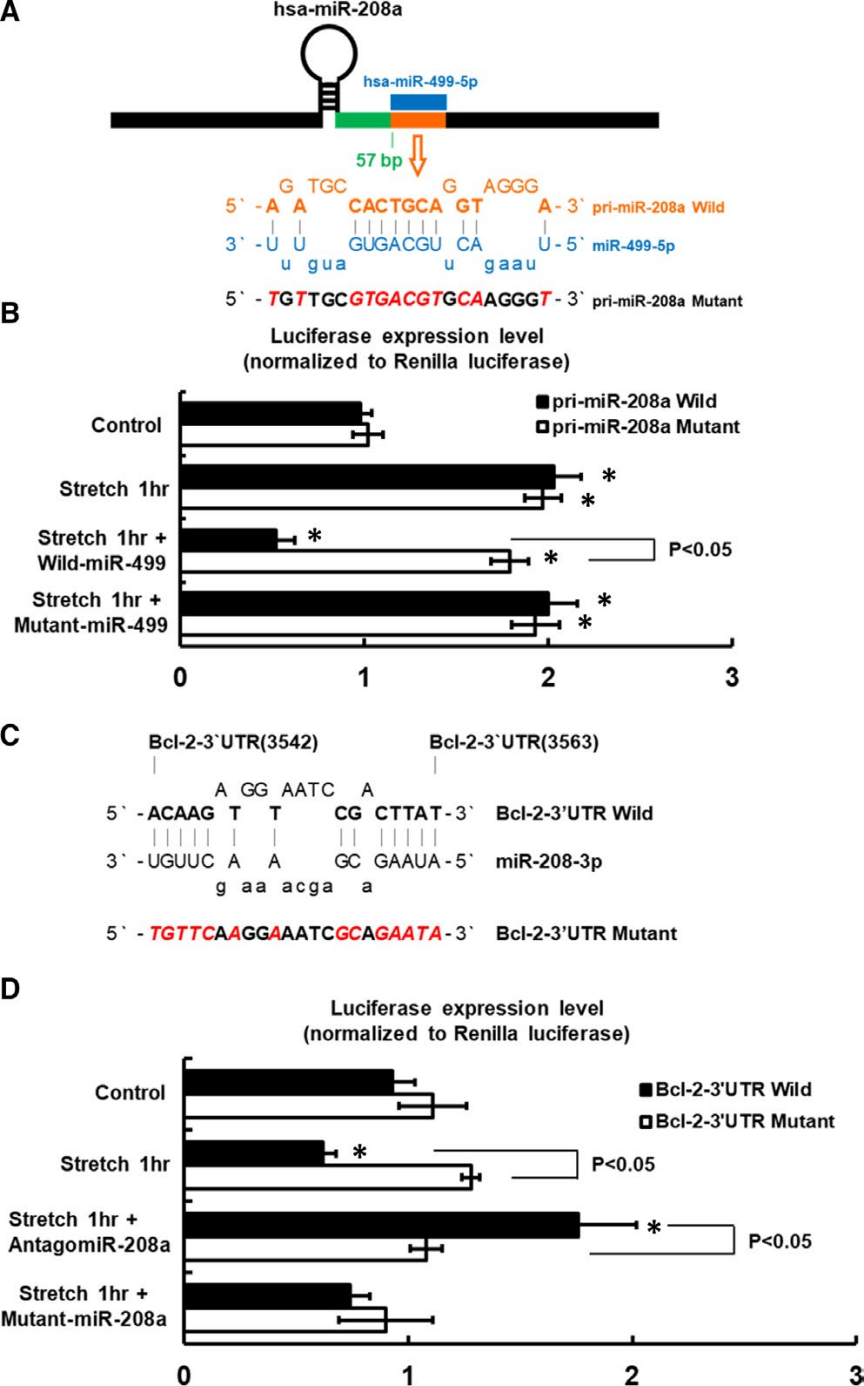
miR‐499 overexpression inhibits pri‐miR‐208a luciferase activity under mechanical stress, whereas pre‐treatment with antagomir‐208a enhanced Bcl‐2‐3′UTR luciferase activity. A, Putative binding site for miR‐499‐5p on human pri‐miR‐208a transcript as predicted by luciferase assay. As shown in the schematic diagram, a pri‐miR‐208a matching site (orange rectangle) for miR‐499‐5p (blue rectangle) was located 57bp downstream of the pri‐miR‐208a gene loci. B, Quantitative analysis of wild‐type and mutant pri‐miR‐208a luciferase activity under mechanical stress with miR‐499 and mutant miR‐499. C, Sequence of the Bcl‐2‐3′UTR target site for miR‐208a binding, located at Bcl‐2‐3′UTR (nucleotide from 3542 to 3563). D, Quantitative analysis of wild‐type and mutant Bcl‐2‐3′UTR luciferase activity under mechanical stretching with antagomir‐208a and mutant miR‐208a. Luciferase activity in the cell lysates was measured and normalized by Renilla activity using a dual‐luciferase assay system (n = 5 per group). *P < .001 vs. control

We discovered that human pri‐miR‐208a, located 57bp downstream of the pri‐miR‐208a gene loci, had a binding site for miR‐499‐5p (Figure 2A). Mechanical stretching for 1 h significantly increased pri‐miR‐208a luciferase activity in the HCF‐aa (Figure 2B). However, pre‐treatment with miR‐499 inhibited the expression of pri‐miR‐208a luciferase activity in stretched HCF‐aa. Mutation of the miR‐499‐binding site on pri‐miR‐208a abolished the inhibition of pri‐miR‐208a luciferase activity by stretching for 1 h. The addition of mutant miR‐499 does not affect the luciferase activity. These findings indicated that pri‐miR‐208a is the target of miR‐499.

### 3.3 Mechanical stretching suppressed Bcl‐2‐3’UTR luciferase activity via miR‐208a

The Bcl‐2‐3′UTR contained the miR‐208a binding site (Figure 2C), whereas the mutant Bcl‐2‐3′UTR had a mutation of the miR‐208a binding site. The luciferase reporter assay revealed that mechanical stretching for 1 h inhibited Bcl‐2‐3′UTR luciferase activity (Figure 2D). However, this effect was inhibited by the mutation of the miR‐208a binding site in the Bcl‐2‐3′UTR. The addition of antagomir‐208a enhanced Bcl‐2‐3′UTR luciferase activity in stretched HCF‐aa, but it had no effect on the mutant Bcl‐2‐3′UTR. The addition of mutant miR‐208a had no effect on Bcl‐2‐3′UTR luciferase activity. These results suggest that the miR‐208a binding site in the Bcl‐2‐3′UTR is essential for the transcriptional regulation induced by mechanical stretching.

### 3.4 MiR‐499 inhibited miR‐208a expression in stretched HCF‐aa

**FIGURE 3:**
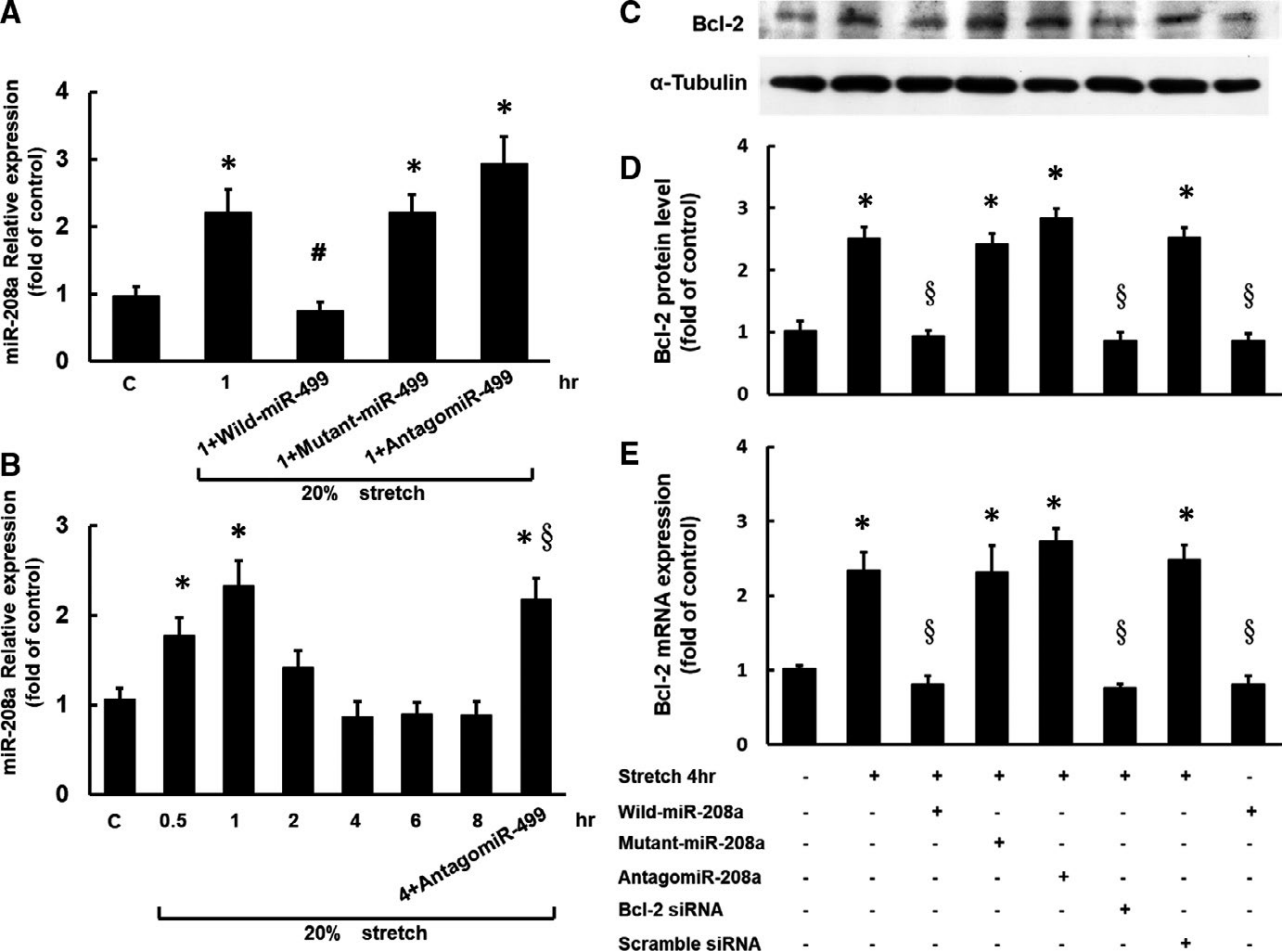
miR‐499 affected the expression of Bcl‐2 mRNA and protein via miR‐208a in stretched human cardiac fibroblasts‐adult atrial (HCF‐aa). A, miR‐208a expression in the HCF‐aa subjected to 20% mechanical stretching for 1 h with the overexpression of miR‐499, mutant miR‐499 and antagomir‐499. B, miR‐208a expression in the HCF‐aa subjected to 20% mechanical stretching for 0‐8 h with the antagomir‐499 overexpression in the HCF‐aa subjected to stretching for 4 h. *P < .001 vs. control. C, Representative Western blot for Bcl‐2 protein expression in the HCF‐aa subjected to stretching at 20% elongation for 4 h with the overexpression of miR‐208a, mutant miR‐208a, antagomir‐208a, Bcl‐2 small interfering RNA (siRNA) and scramble siRNA. D, E, Quantitative analysis of Bcl‐2 protein and mRNA levels in stretched HCF‐aa with the overexpression of miR‐208a, mutant miR‐208a, antagomir‐208a, Bcl‐2 siRNA and scramble siRNA. (n = 3 per group) *P < .001 vs. control or no treatment, # *P* < .001 vs. stretch for 1 hr, §*P* < .001 vs. stretch for 4 hr

Compared with stretching alone, the miR‐499 overexpression significantly inhibited the miR‐208a expression in the HCF‐aa that had been subjected to mechanical stretching for 1 h (Figure 3A). However, the miR‐208a expression was not inhibited by the addition of mutant miR‐499 or antagomir‐499. The addition of antagomir‐499 attenuated the miR‐208a down‐regulation with stretching for 4 h (Figure 3B). These findings suggested that the down‐regulation of miR‐208a expression may have resulted from the miR‐499 up‐regulation during mechanical stretching.

### 3.5 Mechanical stretching affected the expression of Bcl‐2 mRNA and protein via miR‐208a

Compared with stretching alone, the addition of miR‐208a significantly inhibited the expression of Bcl‐2 mRNA and protein induced by mechanical stretching for 4 h (Figure 3C–E), whereas the addition of mutant miR‐208a or antagomir‐208a had no effect on this Bcl‐2 expression. Compared with stretching alone, Bcl‐2 small interfering RNA (siRNA) inhibited the expression of Bcl‐2 mRNA and protein induced by mechanical stretching for 4 h, whereas the addition of scrambled siRNA had no effect on this inhibitory effect. Pre‐treatment with wild‐type miR‐208a has no effect on the Bcl‐2 expression in the HCF‐aa without stretching.

**FIGURE 4:**
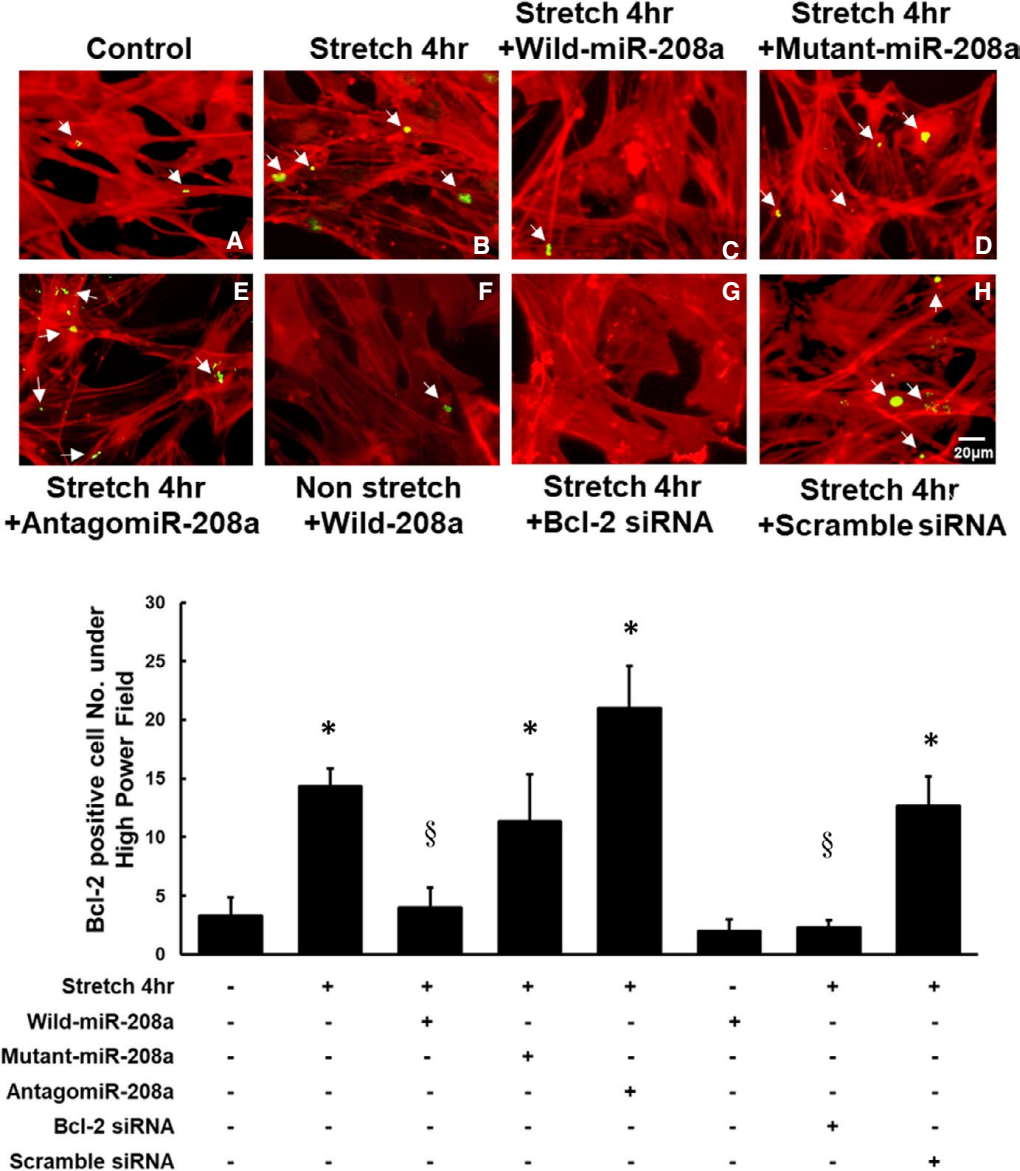
Immunohistochemical staining confirmed the Bcl‐2 expression in human cardiac fibroblasts‐adult atrial (HCF‐aa). Confocal microscopy showing immunoreactive signals of Bcl‐2 (white arrow) in the HCF‐aa under the following conditions (n = 3 per group): Upper panel, A, Control. B, Mechanical stretching to 20% elongation for 4 h. C, Stretching for 4 h with miR‐208a overexpression. D, Stretching for 4 h with mutant miR‐208a overexpression. E, Stretching for 4 h with antagomir‐208a. F, miR‐208a overexpression in the HCF‐aa without mechanical stretching. G, Stretching for 4 h with the addition of Bcl‐2 siRNA. H, Stretching for 4 h with scramble siRNA. Lower panel, Quantitative analysis of the immunohistochemical staining results. *P < .001 vs. control, § *P* < .001 vs. stretch for 4 h

Immunohistochemical staining confirmed the increased Bcl‐2 expression in the HCF‐aa after mechanical stretching for 4 h (Figure 4A,B). The addition of wild‐type miR‐208a inhibited this Bcl‐2 expression, whereas pre‐treatment with mutant miR‐208a or antagomir‐208a had no effect (Figure 4C–E). Wild‐type miR‐208a has no effect on the Bcl‐2 expression in the HCF‐aa without stretching (Figure 4F). The addition of Bcl‐2 siRNA inhibited the Bcl‐2 expression in the HCF‐aa after stretching for 4 h, whereas the addition of scrambled siRNA had no effect on this inhibition (Figure 4G,H). These results suggest that mechanical stretching for 4 h affects the Bcl‐2 expression via miR‐208a.

### 3.6 Mechanical stretching‐induced apoptosis was mediated by miR‐499 and miR‐208a in HCF‐aa

**FIGURE 5:**
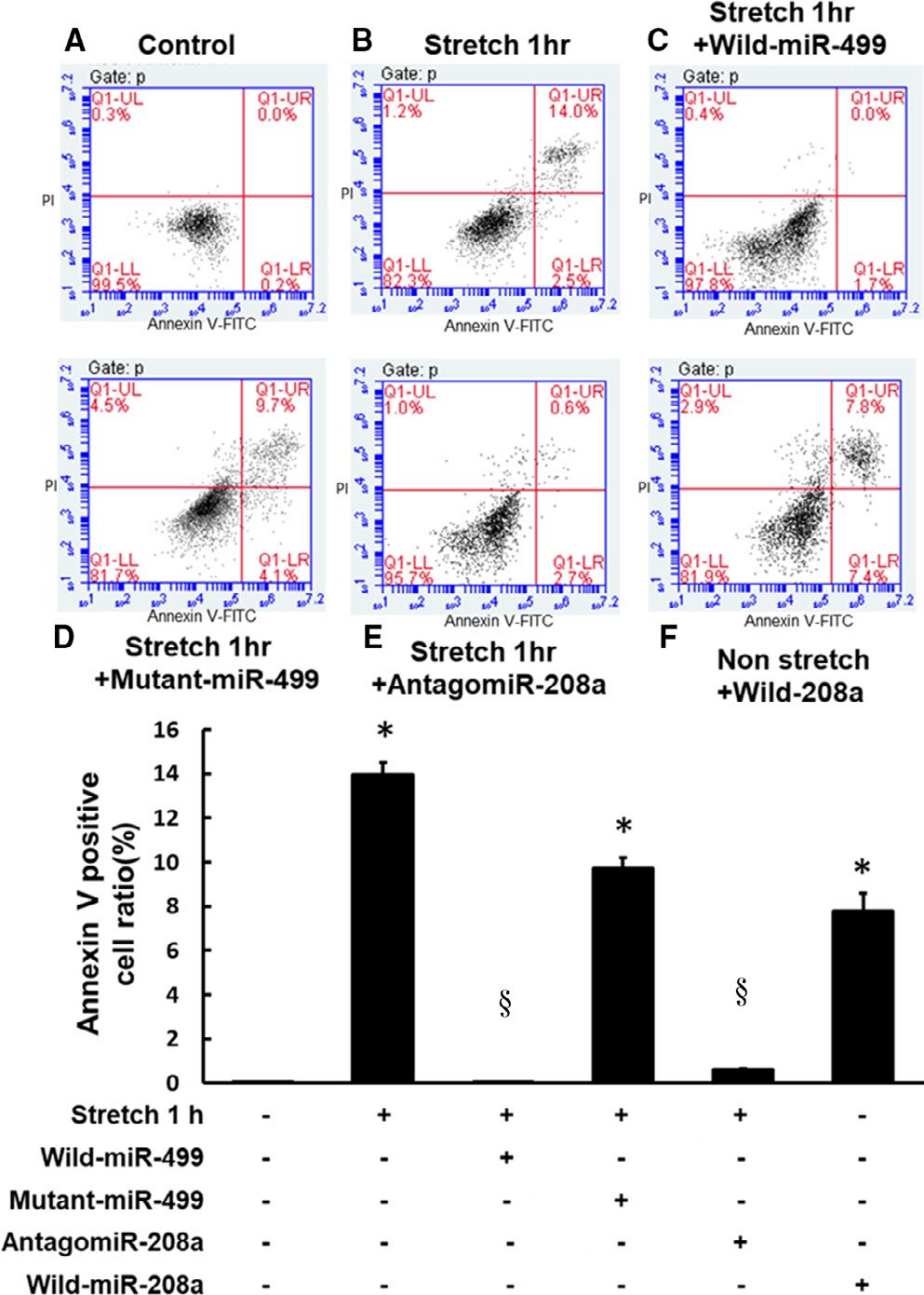
Fluorescein isothiocyanate‐annexin V analyses of human cardiac fibroblasts‐adult atrial (HCF‐aa) under various conditions. Cells that stained negative for both annexin V and propidium iodide were alive, those that stained positive for annexin V and negative for propidium iodide were undergoing apoptosis, and those that stained positive for both annexin V and propidium iodide were in the end stage of apoptosis (second apoptosis). Upper panel, A, Control. B, Mechanical stretching for 1 h. C, Mechanical stretching for 1 h with miR‐499. D, Mechanical stretching for 1 h with mutant miR‐499. E, Mechanical stretching for 1 h with antagomir‐208a. F, miR‐208a without mechanical stretching. FACScan was used to quantify the apoptotic fraction. Lower panel, Quantitative analysis of the percentage of cells stained with both propidium iodide and annexin V. (n = 4 per group). *P < .001 vs. control, §*P* < .001 vs. stretch for 1 h

Propidium iodide‐annexin V double‐staining and FACS analysis were used to evaluate apoptosis. The percentage of cells stained with both propidium iodide and annexin V after stretching for 1 h was significantly higher than in the control group (Figure 5A,B). The observed increases in propidium iodide and annexin V staining were significantly reversed by the miR‐499 overexpression, whereas mutant miR‐499 had no effect on apoptosis (Figure 5C,D). Pre‐treatment with antagomir‐208a inhibited apoptosis after mechanical stretching for 1 h, whereas the wild‐type miR‐208a overexpression enhanced apoptosis in the HCF‐aa that had not undergone mechanical stretching (Figure 5E,F). Figure 5 lower panel showed the apoptotic quantification results of cells stained with both annexin V and propidium iodide. These results indicate that both miR‐499 and miR‐208a play an essential role in the HCF‐aa apoptosis after mechanical stretching.

### 3.7 MiR‐499 regulated myocardial Bcl‐2 expression via miR‐208a in rats with AV shunts

**FIGURE 6:**
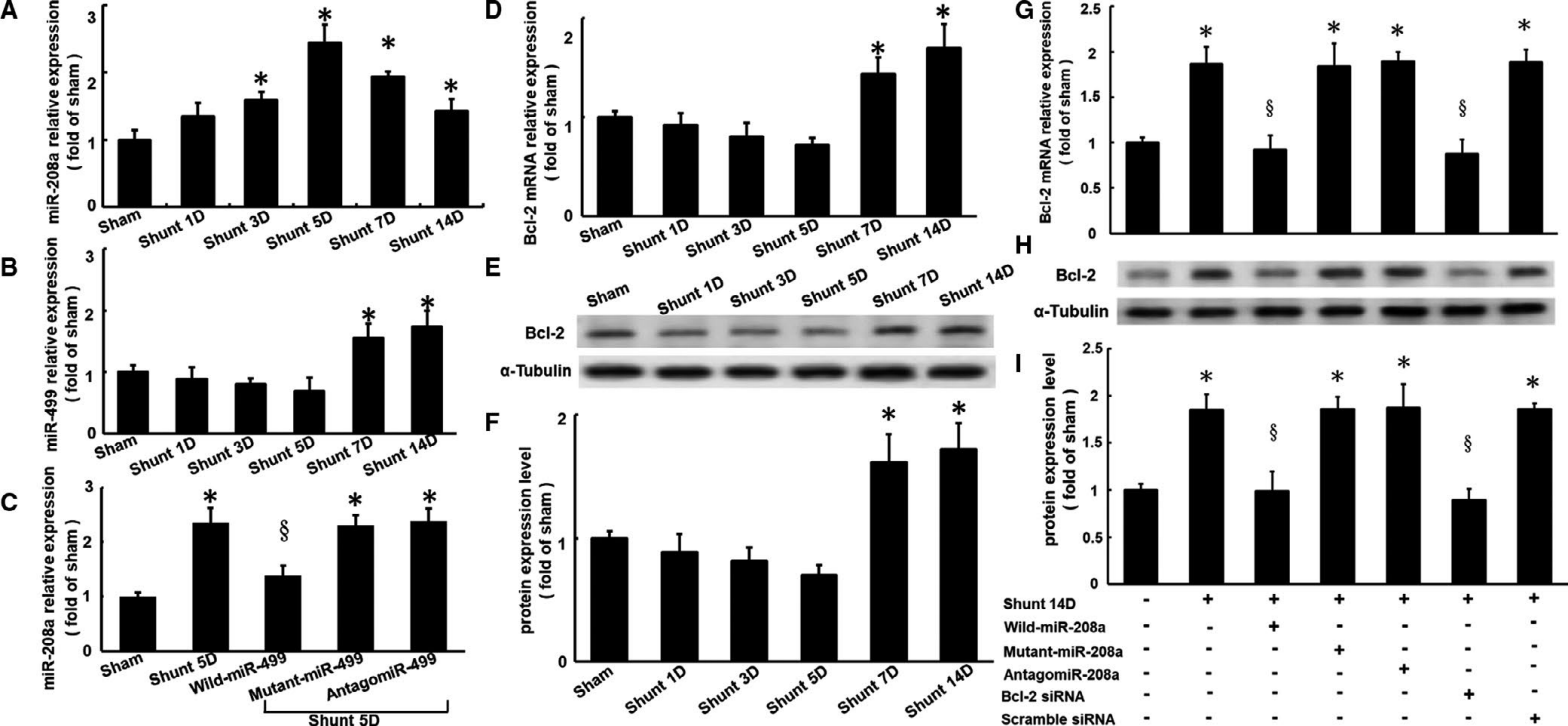
miR‐499 regulated myocardial Bcl‐2 expression via miR‐208a in rats with AV shunts. Representative expression of miR‐208a (A) and miR‐499 (B) in the left ventricular myocardium of rats with an aortocaval (AV) shunt for 1‐14 days (D). C, miR‐208a expression in rats with AV shunts for 5 days with the addition of miR‐499, mutant miR‐499 and antagomir‐499. D, Representative expression of Bcl‐2 protein for 1‐14 days in rats with an AV shunt. E, F, Representative Western blot and quantitative analysis, respectively, of Bcl‐2 expression in the left ventricular myocardium of rats with an AV shunt. *P < .001 vs. stretching. G, Representative Bcl‐2 mRNA expression. H, I, Western blot and quantitative analysis of Bcl‐2 expression in the rat myocardium after volume overload by an AV shunt for 14 days with the addition of miR‐208a, mutant miR‐208a, antagomir‐208a, Bcl‐2 siRNA and scramble siRNA (n = 4 per group). *P < .001 vs. sham, *P* < .001 vs. shunt for 5D or 14D

The influence of miR‐499 and miR‐208a on Bcl‐2 expression in vivo was examined in adult rats with aortocaval (AV) shunts to induce volume overload. The haemodynamic and echocardiographic parameters of the failing heart induced by AV shunt were summarized in Table S1. The AV shunt resulted in a significant increase in myocardial miR‐208a expression from 3 to 5 days but decreased from 7 to 14 days (Figure 6A). Additionally, miR‐499 expression increased from 7 to 14 days (Figure 6B). The wild‐type miR‐499 overexpression significantly reduced the miR‐208a expression at 5 days, but the addition of mutant miR‐499 and antagomir‐499 had no effect on miR‐208a expression (Figure 6C).

Rats with AV shunts exhibited significant induction of myocardial Bcl‐2 mRNA and protein expression from 7 to 14 days compared with sham rats (Figure 6D–F). After 14 days with an AV shunt, the rats exhibited a significant increase in myocardial Bcl‐2 mRNA and protein expression (Figure 6G–I). Pre‐treatment with miR‐208a significantly inhibited this increase, whereas the addition of mutant miR‐208a and antagomir‐208a had no effect. Pre‐treatment with Bcl‐2 siRNA inhibited the expression of myocardial Bcl‐2 mRNA and protein compared with the rats with AV shunts, whereas the addition of scrambled siRNA had no effect on this inhibition. These findings suggested that the miR‐208a expression could be regulated by miR‐499 and may regulate Bcl‐2 expression in rat hearts with volume overload.

## 4 DISCUSSION

The major findings of this study were as follows: (a) mechanical stretching or an AV shunt placement first up‐regulated and later down‐regulated miR‐208a expression in the HCF‐aa or the rat myocardium; (b) mechanical stretching or an AV shunt placement up‐regulated miR‐499 and Bcl‐2 expression in the HCF‐aa or rat myocardium with volume overload; (c) the miR‐499‐5p binding site in the pri‐miR‐208a and the miR‐208a‐3p binding site in the Bcl‐2‐3′UTR were essential for the transcriptional regulation induced by mechanical stretching; (d) miR‐499 inhibited the miR‐208a expression in stretched HCF‐aa and rat myocardium with volume overload; (e) the Bcl‐2 expression was suppressed by miR‐208a in the HCF‐aa during stretching and in the rat myocardium with an AV shunt; (f) the overexpression of miR‐499 or antagomir‐208a attenuated cellular apoptosis in stretched HCF‐aa.

Mechanical stretching promotes cardiac remodelling by increasing apoptosis.^18^ Recent studies suggest that mechanical stretching simultaneously causes cardiomyocyte apoptosis and cardiac fibroblast proliferation, resulting in reduced myocardial contractility and increased fibrous tissue.^18^, ^19^, ^20^ Cardiomyocyte fibrosis is a critical event in cardiac remodelling and may evolve into heart failure. It has been reported that miRNA 1, 29b, 34a, 101, 122, 208a and 320 play a role in cardiomyocyte apoptosis, whereas miRNA 21, 30, 125b, 133 and 206 have antiapoptotic effects.^21^ MicroRNAs are non‐coding RNAs with 20‐25 nucleotides in length that transcriptionally or post‐transcriptionally modulate the presentation of their target gene.^22^ Current strategies of therapeutic cardiovascular diseases are targeted towards regenerative therapies; the combination of miR‐1, miR‐133, miR‐208 and miR‐499 can regulate cardiomyocyte regeneration.^23^ However, the effects of miR‐499 on the regulation of miR‐208a expression in atrial fibroblasts are currently unknown. In previous studies, microRNA regulation by other microRNA has been almost ignored when studying the mechanism of microRNA effects. Such regulation of one microRNA by another has rarely been reported in studies on physiologic and pathologic mechanisms of microRNA effects. Indeed, there are several examples of microRNA‐mediated microRNA regulation within the family of myomiRs, which seems to be considered as a broader and more general function of microRNAs. Using the transgenic expression of miR‐499 in mouse heart, Matkovich et al reported that miR‐499 indirectly regulated hundreds of mRNAs and approximately 17 cardiac microRNAs expression, including down‐regulation of miR208a in rat cardiomyocytes.^6^ Interestingly, van Rooij et al demonstrated that miR‐499 is significantly extinguished in the heart with miR‐208a knockout mice.^5^ As schematized in Figure 1, an early miR‐208a up‐regulation was observed in HCF‐aa under mechanical stretch for 1 h, followed by a miR‐499 increase and a later miR‐208a decrease at 4 h of stretching. Besides, pre‐treatment with miR‐499 down‐regulated miR‐208a level at an early mechanical stretch, while adding antagomir‐499 up‐regulated miR‐208a level at 4 h of stretching (Figure 3A,B). Taken together, these findings might suggest that miR‐499 down‐regulated miR‐280a level at the late phase of the present study, and its up‐regulation by miR‐208a, noticed by Rooij et al's finding, might be protective negative feedback.

Accumulating evidence indicated that miR‐133, miR‐1 and miR‐499 were involved in the differentiation of progenitor cell into cardiomyocytes; furthermore, by cycling contraction of mouse P19 cells, the combination of miR‐499 and miR‐133 exerted a synergic effect on cardiac differentiation.^24^ Those investigations indicated that microRNAs might mediate or have a synergic effect on another microRNA to generate a certain function. MiR‐208a is reported to play a pathological role in cardiac hypertrophy and consequently arrhythmia.^25^ MiR‐499, encoded by intron of Myh7 together with miR‐208b, and miR‐208a, belonging to Myh6, were recently suggested to be an important regulator of the genetic network in the differentiation of stem cells into cardiomyocytes.^5^, ^26^ The present study indicated that miR‐208a was significantly up‐regulated in the HCF‐aa that had been subjected to mechanical stretching for 1 h and that the miR‐208a expression was suppressed by the miR‐499 overexpression. The luciferase activity analysis verified that the removal of the miR‐499‐5p binding site in the pri‐miR‐208a area abolished the suppressive effect of miR‐499 on pri‐miR‐208a luciferase activity in stretched HCF‐aa. These findings suggest that the reduction of the pri‐miR‐208a expression determined by mechanical stretching was dependent on miR‐499. Therefore, it is likely that enhanced miR‐499 expression associated with mechanical stretching down‐regulates miR‐208a expression. Taken together, miR‐499 that modulates cardiac transcription can directly regulate miR‐208a, which indicates that microRNAs that modulate cardiac transcription can regulate other microRNAs.

The Bcl‐2 protein principally determines whether a cell commits to apoptosis and hence has a crucial role in cellular development. Consequently, the role of Bcl‐2 protein has the potential role to affect multiple mechanism of cardiomyocyte apoptosis in response to various stress, including hypoxia, calcium dysregulation and oxidative stress.^27^, ^28^ The relationship between Bcl‐2 and miR‐208a was rarely reported in the past. In the present study, the Bcl‐2 mRNA expression level after 1 h stretching was significantly lower than controls, while up‐regulation of miR‐208a at 1 h stretching. The addition of antagomir‐208a reversed the inhibition of Bcl‐2 expression at 1 h stretching (Figure 2D). Besides, levels of Bcl‐2 mRNA and protein were consistently increased after 2‐8 h of mechanical stretching, whereas miR‐208a was down‐regulated. These findings were consistent with previous observations that Bcl‐2 expression increased under physiological stressful conditions, such as oxidative stress^29^ and hypoxia,^30^ and that the expression of Bcl‐2 could attenuate cardiomyocyte apoptosis.^31^ Additionally, the present study found that pre‐treatment with miR‐208a inhibited Bcl‐2 mRNA and protein expression in the HCF‐aa under mechanical stretching for 4 h, whereas the addition of antagomir‐208a abolished the suppression of the transcriptional activity of Bcl‐2 in stretched HCF‐aa and thereby inhibited cellular apoptosis in the HCF‐aa under mechanical stretching. Besides, Matkovich et al group also observed that Bcl‐2 mRNA up‐regulation in transgenic miR‐499 expression mice.^6^ Taken together, the suppression of miR‐208a expression by miR‐499 resulted in the relief of the suppression of Bcl‐2, thereby avoiding HCF‐aa apoptosis. The mediation of microRNA regulation by another microRNA greatly complicated and amplified microRNA‐mediated signal redirection and phenotypic outcomes.^4^, ^5^

**FIGURE 7:**
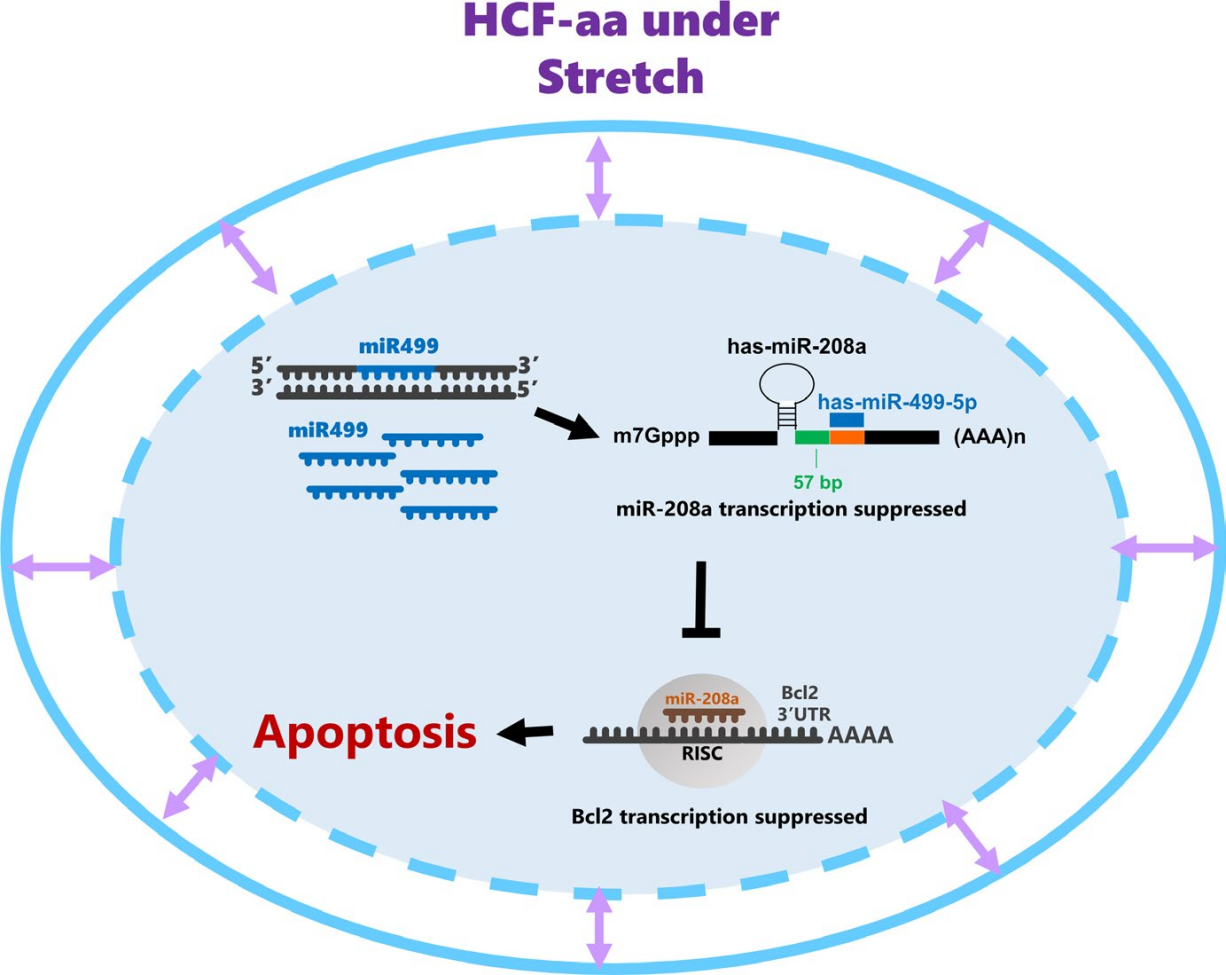
Proposed pathway of miR‐208a mediated the Bcl‐2 expression by miR‐499 in human cardiac fibroblasts‐adult atrial (HCF‐aa). The critical role of miR‐499 might bring a potential application for microRNA‐directed therapy of cardiovascular disease

In conclusion, we found that miR‐208a was a direct target of miR‐499 and that the suppression of miR‐208a relieved the suppression of Bcl‐2 in mechanically stretched HCF‐aa (Figure 7). We also observed that antagomir‐208a overexpression enhanced Bcl‐2 expression in a model of heart failure induced by volume overload. The results of microRNA‐mediated microRNA regulation are a potential new application of microRNA‐directed cardiovascular therapy.

## CONFLICT OF INTERESTS

The authors declare no conflict of interests.

## AUTHOR CONTRIBUTIONS

SKC: Study design and manuscript draft. BWW, YJY and WJF: Most of the experiments. CML and KGS: Study design and manuscript revision. All authors provided clarification and guidance on the manuscript, were involved in editing the manuscript and approved the final manuscript.

## ETHICS APPROVAL AND CONSENT TO PARTICIPATE

All animal procedures were performed in accordance with the Institutional Committee of Animal Care and Use (Protocol number #0990816001) and conformed to the Guide for the Care and Use of Laboratory Animals published by the US National Institutes of Health (NIH publication No. 86‐23, revised 1996).

## CONSENT FOR PUBLICATION

Not applicable.

## Supporting information

Table S1

## Data Availability

All data generated or analysed during this study are included in this published article.
