# Supplementary material for: Cyclic stretching boosts microRNA‐499 to regulate Bcl‐2 via microRNA‐208a in atrial fibroblasts
Source: J Cell Mol Med. 2021 Feb 18;25(6):3113–23. doi: 10.1111/jcmm.16373 (PMC7957261; doi:10.1111/jcmm.16373)
Supplement: Supplementary file 1 — Table S1 [file JCMM-25-3113-s001.docx]

**Supplementary Table. Hemodynamic and echocardiographic parameters of the failing heart induced by aorta-caval shunt**

|  | Sham | Shunt 7D | Shunt 14D | Shunt 14D + Antagomir-208a | Shunt 14D + Mut-208a | Shunt 14D + miR-499 | Shunt 14D + Mut-499 |
| --- | --- | --- | --- | --- | --- | --- | --- |
| N | 4 | 4 | 5 | 4 | 4 | 4 | 3 |
| Body weight, g | 329 ± 21 | 310 ± 22 | 298 ± 27 | 304 ± 17 | 292 ± 23 | 320 ± 27 | 301 ± 29 |
| Heart weight, mg | 810 ± 33 | 905 ± 46 | 1073 ± 61* | 950 ± 43^§^ | 1053 ± 44* | 946 ± 32^§^ | 1049 ± 37* |
| Heart weight/body weight, mg/g | 2.5± 0.6 | 2.9 ± 0.9 | 3.3 ± 0.6 | 2.9 ± 0.7 | 3.4 ± 0.9* | 3.0 ± 0.8 | 3.5 ± 0.4 |
| Heart rate, min | 332 ± 36 | 359 ± 47 | 374 ± 52 | 342 ± 42 | 362 ± 38 | 350 ± 42 | 359 ± 48 |
| MAP, mmHg | 115 ± 14 | 88 ± 13 | 64 ± 10* | 81 ± 11 | 62 ± 12* | 91 ± 16^§^ | 72 ± 12* |
| LVEDD, mm | 6.5 ± 0.6 | 7.55 ± 0.9 | 8.25 ± 0.7* | 7.2 ± 0.8 | 7.98 ± 0.5* | 7.26 ± 0.4 | 7.91 ± 0.5* |
| LVESD, mm | 3.02 ± 0.5 | 4.46 ± 0.8 | 5.31 ± 0.9* | 3.97 ± 0.7^§^ | 5.2 ± 0.7* | 3.72 ± 0.8^§^ | 5.02 ± 0.8* |
| FS, % | 52 ± 9 | 42 ± 8 | 37 ± 6* | 46 ± 8 | 39 ± 9* | 47 ± 8 | 37 ± 9* |

*p<0.05 vs. Sham; ^§^ p< 0.05 vs. Shunt 14D

MAP =mean arterial pressure, LVPWT = left ventricular posterior wall thickness, LVEDD = left ventricular end-diastolic dimension, LVESD = left ventricular end-systolic dimension, FS = fraction shortening.
